# Robust Random Walk Based on Natural Neighbors for Outlier Detection

**DOI:** 10.3390/e28070734

**Published:** 2026-06-29

**Authors:** Ken Chen, Wenyao Zhu, Tiansong Li, Hongkui Wang

**Affiliations:** 1 College of Artificial Intelligence, Lishui University, Lishui 323000, China; 2 Zhejiang Rongqe Technology Co., Ltd., Lishui 323000, China; 3 School of Computer and Information Science, Chongqing Normal University, Chongqing 401331, China; 4 Lishui Institute, Hangzhou Dianzi University, Lishui 323000, China

**Keywords:** granular computing, shadowed sets, natural neighbor, outlier detection, random walk

## Abstract

Outlier detection serves as an effective technique for identifying anomalous samples in complex data. Existing methods are often disturbed by noise and boundary samples, which degrade the quality of sample relationships. Moreover, traditional random walk approaches are vulnerable to weak and spurious connections that can mislead the walking process. To address these issues, this paper proposes a robust random walk based on natural neighbors for outlier detection (RWNOD) method. First, an adaptive smoothing mechanism is proposed to leverage natural neighbors to actively adjust sample positions, reducing local noise while preserving structural information. Then, a robust random walk strategy is developed to incorporate shadowed sets into the transition matrix, preserving reliable connections while suppressing unreliable ones. At the same time, a corresponding outlier detection algorithm is proposed. Experiments on datasets are conducted to compare the proposed algorithm with seven other algorithms. The experimental results demonstrate that the proposed algorithm achieves superior performance and strong robustness.

## 1. Introduction

Outlier detection serves as an essential machine learning technique for recognizing samples that significantly diverge from mainstream patterns. As a fundamental preprocessing step, effective outlier removal can substantially improve the performance of downstream learning models. Beyond data preprocessing, outlier detection techniques have been widely deployed in numerous practical scenarios, including financial auditing [1], industrial quality control [2], healthcare analytics [3], network security [4], spatio-temporal anomaly detection [5], maritime traffic monitoring [6], and pharmaceutical cold chain logistics [7]. Therefore, developing advanced outlier detection algorithms holds considerable practical value.

Relationships among samples constitute a critical foundation for outlier detection, as the quality of these relationships directly influences the discriminability between normal and anomalous instances. Considerable research efforts have consequently been devoted to optimizing sample relationships [8,9,10,11]. Xia et al. [12] optimized neighborhood relationships by adaptively generating granular-balls of varying sizes. Yuan et al. [13] enhanced fuzzy relationships through weighted fuzzy-rough density. Wang et al. [14] assigned weights to neighbor samples based on standard deviation to mitigate noise sensitivity in neighborhood relationships. Zhang et al. [15] captured complex structural relationships using bi-level granular-ball knowledge representation. Su et al. [16] defined a local distribution-aware fuzzy relation that incorporates local data distribution into similarity calculations, enabling more discriminative fuzzy relationships between samples. Zhang et al. [17] dynamically adjusted granular relationships based on local data distribution. In parallel, Zhang et al. [18] proposed ODMGIS to capture data distribution patterns through multi-granularity information sets, while Deng et al. [19] developed an outlier detection method based on multiple information extraction by integrating neighborhood-weighted entropy and fuzzy difference-ratio entropy. However, these methods optimize relationships passively within the original data space, either by weighting or by adaptive neighborhood generation, without actively adjusting the data distribution itself. Noise and boundary samples continue to degrade relationship quality, as the underlying data positions remain unchanged. Therefore, the **adaptive smoothing mechanism** is proposed to leverage natural neighbors to actively adjust sample positions, reducing local noise while preserving structural information.

Random walk has been widely adopted as an effective tool for outlier detection by analyzing transition patterns within graph-based data representations. Moonesinghe et al. [20] pioneered the use of random walks on similarity graphs, treating nodes with low visitation frequencies as outliers. Chun et al. [21] extended random walk with restart to hypergraphs, enabling anomaly detection on complex relational structures. To strengthen outlier separability, Wang et al. [22] introduced virtual nodes into the graph to amplify abnormal signals. For heterogeneous data, Wang et al. [23] developed a weighted neighbourhood information network framework that integrates attribute-aware neighborhood analysis. Liu et al. [24] advanced the paradigm by embedding fuzzy granular computing into anomaly scoring to handle uncertain relational patterns. Recently, Wang et al. [25] incorporated granular-ball computing into random walks to enable efficient multi-scale outlier detection. Zhang et al. [15] further extended this direction by integrating bi-level granular-ball knowledge representation with second-order biased random walks. However, these random walk methods construct transition relationships directly from raw data, making them vulnerable to weak and noisy connections that can mislead the walking process. Even after smoothing, unreliable connections may persist in the similarity matrix, obscuring the underlying data structure. To overcome this limitation, the **robust random walk strategy** is developed to incorporate shadowed sets into the transition matrix, preserving reliable connections while suppressing unreliable ones.

In view of the above analysis and investigation, this paper proposes an outlier detection model based on the adaptive smoothing mechanism and the robust random walk strategy. Therefore, the main contributions of this paper are as follows.

1. An adaptive smoothing mechanism is proposed to leverage natural neighbors to actively adjust sample positions, reducing local noise while preserving structural information.
2. A robust random walk strategy is developed to incorporate shadowed sets into the transition matrix, enhancing structural awareness by preserving reliable connections and suppressing unreliable ones.
3. A robust random walk based on natural neighbors for outlier detection method is proposed, and the corresponding outlier detection algorithm is developed.
4. Experimental results demonstrate that the proposed algorithm outperforms state-of-the-art outlier detection methods in both accuracy and robustness.

The remainder of this paper is organized as follows. Section 2 introduces the preliminary knowledge on shadowed sets, natural neighbors, and Markov random walk. Section 3 presents the adaptive smoothing mechanism and the robust random walk strategy. In Section 4, we develop the RWNOD outlier detection algorithm and analyze its time complexity. The results of our experiments are shown in Section 5. Finally, Section 6 summarizes this paper.

## 2. Preliminaries

### 2.1. Shadowed Sets

Shadowed sets provide a three-way decision representation tool within the framework of fuzzy set theory [26]. By transforming fuzzy memberships into three distinct regions, they offer an effective mechanism for handling uncertainty and boundary fuzziness in data.

**Definition** **1.** *An information system can be formalized as a quadruple S=(U,A,V,f), where:*

- *$U=\{u_{1},u_{2},\cdots ,u_{n}\}$ is a nonempty finite set of objects;*
- *$A=\{a_{1},a_{2},\cdots ,a_{m}\}$ is a nonempty finite set of attributes;*
- *$V={⋃}_{a\in A}V_{a}$ denotes the union of value domains for all attributes;*
- *f:U×A→V is an information function that assigns a value $f\left(u,a\right)\in V_{a}$ to each object-attribute pair.*

**Definition** **2.** *Let S=(U,A,V,f) be an information system, and let μ: U→[0,1] be a fuzzy membership function defined on U. For a given pair of threshold parameters (α,β) satisfying 0≤α<β≤1, the shadowed set mapping $T_{\mu }^{\alpha ,\beta }$:U→{0,[0,1],1} is defined as follows:*

$$T_{\mu }^{\alpha ,\beta }\left(u\right)=\left\{\begin{array}{cc} 1, & \mu (u)\geq \beta ;\\ \mu (u), & \alpha <\mu (u)<\beta ;\\ 0, & \mu (u)\leq \alpha . \end{array}\right.$$

### 2.2. Natural Neighbors

Natural neighbors are based on mutually recognized adjacency, reflecting the true bidirectional relationship between data points [27]. This idea is inspired by the principle of reciprocity in data distribution: if a sample $u_{i}$ considers $u_{j}$ to be a neighbor, then $u_{j}$ should also consider $u_{i}$ as a neighbor. The concept of mutual neighborhood forms the foundation of natural neighbors.

**Definition** **3.** *For any $u_{i}\in U$, let $NN(u_{i})$ denote the set of nearest neighbors of $u_{i}$, and $RNN(u_{i})$ denote the set of reverse nearest neighbors of $u_{i}$. The natural neighbor search process adaptively expands the neighborhood radius until every object has at least one reverse neighbor. Upon satisfying this condition, the natural neighbors of $u_{i}$ are defined as:*

$$N\left(u_{i}\right)=NN\left(u_{i}\right)\cap RNN\left(u_{i}\right).$$

The search algorithm for natural neighbors typically employs a KD-tree structure to accelerate nearest neighbor queries [28], and the overall process is summarized in Algorithm 1. The time complexity of this algorithm is O(|U|log|U|) [29].

### 2.3. Markov Random Walk

Markov random walk is a fundamental stochastic process widely used for modeling sequential state transitions [30,31]. It is formally defined as a discrete-time stochastic process ${\left\{X_{t}\right\}}_{t=0,1,\ldots }$ that adheres to the Markov property: the probability distribution of the subsequent state $X_{t}$ depends only on the current state $X_{t-1}$, i.e.:

(1) $$P\left(X_{t}|X_{0},X_{1},\ldots ,X_{t-1}\right)=P\left(X_{t}|X_{t-1}\right).$$

**Algorithm 1:** Natural neighbor searching algorithm
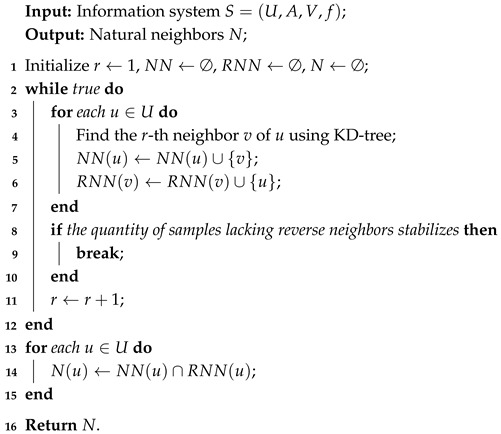


For a chain with *n* states, the transition probabilities are encoded in a matrix $P\in R^{n\times n}$, where $p_{ij}$ denotes the probability of transitioning from state *i* to state *j*. Under mild regularity conditions, the state distribution converges to a unique stationary distribution $\pi \in R^{n}$ satisfying:

(2) π=πP.

The stationary distribution characterizes the long-term visiting frequency of each state, providing a theoretical foundation for graph-based outlier detection.

## 3. Robust Random Walk Based on Natural Neighbors for Outlier Detection Model

This section presents the proposed robust random walk based on natural neighbors for outlier detection (RWNOD) model. The core of our approach integrates two synergistic components: the **adaptive smoothing mechanism** is proposed to leverage natural neighbors to reduce local noise and preserve structural information; and the **robust random walk strategy** is designed to incorporate shadowed sets into the transition matrix, enhancing structural awareness. This design ensures that the model is capable of detecting outliers robustly and effectively. The following subsections provide detailed expositions of these two key components.

### 3.1. Adaptive Smoothing Mechanism

Natural neighbors provide a parameter-free approach for characterizing local data distributions by capturing bidirectional adjacency relationships. However, the presence of noise and outliers can interfere with the accurate identification of natural neighbor relationships, potentially compromising the quality of subsequent analysis. Therefore, the **adaptive smoothing mechanism** is proposed to leverage natural neighbors to reduce local noise and preserve structural information.

**Definition** **4** (Gaussian Kernel Weight)**.** *For any $u_{i}\in U$ and $u_{j}\in N\left(u_{i}\right)$, the weight is defined as:*

(3) $$w_{ij}=\exp\left(-\frac{∥u_{i}-u_{j}{∥}^{2}}{2\sigma_{s}^{2}}\right),$$

*where $\sigma_{s}$ is the median of all pairwise distances between samples and their natural neighbors, and $w_{ii}=0$ to exclude the sample’s own influence.*

**Definition** **5** (Smoothed Position)**.** *For any $u_{i}\in U$, the smoothed position ${\tilde{u}}_{i}$ is obtained by adaptively fusing the original sample with its natural neighbors:*

(4) $${\tilde{u}}_{i}=\left(1-\alpha \right)u_{i}+\alpha \frac{\sum_{u_{j}\in N\left(u_{i}\right)}w_{ij}u_{j}}{\sum_{u_{j}\in N\left(u_{i}\right)}w_{ij}},$$

*where α∈[0,1] is the smoothing parameter. If a sample has no natural neighbors, its smoothed position remains unchanged, i.e., ${\tilde{u}}_{i}=u_{i}$, preserving potentially anomalous samples that are isolated from the data distribution.*

The adaptive smoothing mechanism preserves cluster boundaries through three design choices. First, natural neighbor relationships are mutual and bidirectional; samples from different clusters rarely recognize each other as natural neighbors when separated by low-density regions. Second, the Gaussian kernel weights $w_{ij}$ decay exponentially with distance, so contributions from cross-cluster samples are negligible when the inter-cluster distance exceeds the local scale $\sigma_{s}$. Third, the smoothing parameter α<1 retains a component of the original position, anchoring the smoothed position and preventing it from drifting across cluster boundaries. These mechanisms collectively ensure that adjacent clusters remain separated after smoothing while local noise is effectively reduced.

**Figure 1:**
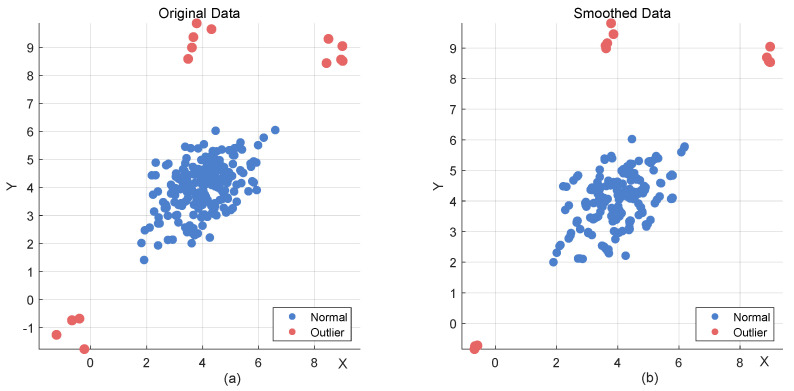
Illustration of the adaptive smoothing mechanism. (**a**) Original data distribution with normal samples (blue) and outliers (red); (**b**) Smoothed data distribution after applying the proposed adaptive smoothing mechanism.

As shown in Figure 1, the adaptive smoothing mechanism effectively reduces local noise while preserving structural information. Normal samples do not simply contract toward a single center; instead, they form locally cohesive substructures by adaptively aggregating with their natural neighbors. Outliers, on the other hand, exhibit noticeable clustering effects among themselves, as they tend to mutually recognize each other as natural neighbors due to their spatial proximity, while remaining largely separated from the normal cluster.

### 3.2. Robust Random Walk Strategy

Traditional random walk methods for outlier detection rely on similarity matrices constructed directly from raw data, which are susceptible to noise and weak connections that can mislead the walking process. While the adaptive smoothing mechanism reduces local noise, the resulting similarity matrix may still contain spurious or unreliable connections. Therefore, the **robust random walk strategy** is developed to incorporate shadowed sets into the transition matrix, enhancing structural awareness.

**Definition** **6** (Shadowed Fuzzy Similarity Relation)**.** *For any $u_{i},u_{j}\in U$, the shadowed fuzzy similarity relation R:U×U→{0,[0,1],1} is defined as:*

(5) $$R\left(u_{i},u_{j}\right)=f_{\delta }\left(\exp\left(-\frac{∥{\tilde{u}}_{i}-{\tilde{u}}_{j}{∥}^{2}}{2\sigma^{2}}\right)\right),$$

*where ${\tilde{u}}_{i}$ and ${\tilde{u}}_{j}$ are the smoothed positions obtained from the adaptive smoothing mechanism, σ is the median of all pairwise distances among smoothed samples, and $f_{\delta }$: [0,1]→{0,[0,1],1} is the shadowed set mapping:*

(6) $$f_{\delta }\left(t\right)=\left\{\begin{array}{cc} 1, & t\geq \delta ;\\ t, & 1-\delta <t<\delta ;\\ 0, & t\leq 1-\delta . \end{array}\right.$$

Unlike hard thresholding that binarizes all connections, the shadowed set mapping preserves the original similarity values in the uncertainty region (1−δ<t<δ). Only high-confidence connections (t≥δ) and confidently absent connections (t≤1−δ) are discretized. This design suppresses weak and spurious connections that may mislead the random walk, while retaining ambiguous information that may be useful for distinguishing subtle structural differences.

**Definition** **7** (Transition Matrix)**.** *Based on the shadowed fuzzy similarity relation R, let $M\in R^{n\times n}$ be the similarity matrix with entries $M_{ij}=R\left(u_{i},u_{j}\right)$. The transition probability matrix $P\in R^{n\times n}$ is constructed by:*

(7) $$P=D^{-1}M,$$

*where $D=\mathrm{diag}(d_{1},d_{2},\ldots ,d_{n})$ is a diagonal matrix with $d_{i}=\sum_{j=1}^{n}M_{ij}$, ensuring each row of P sums to 1. If a row sums to zero, the corresponding row in P is set to a uniform distribution to ensure connectivity.*

*The transition matrix P defines a Markov chain on the sample graph, where the transition probability between samples is proportional to their shadowed fuzzy similarity. This design assigns higher probabilities to transitions toward more similar samples, while suppressing transitions via weak connections.*

*To ensure convergence to a unique stationary distribution, a damping strategy is employed. The update rule is:*

(8) $$\pi^{\left(t+1\right)}=d\cdot u+\left(1-d\right)\cdot \pi^{\left(t\right)}P,$$

*where $\pi^{\left(t\right)}\in R^{n}$ is the probability distribution over samples at iteration t, $u={\left[1/n,\ldots ,1/n\right]}^{⊤}$ is the uniform restart distribution, and d=0.15 is the damping factor based on sensitivity analysis in [25]. When t=0, $\pi^{\left(0\right)}$ is initialized to the uniform distribution. The damping factor d>0 ensures ergodicity by making the transition matrix irreducible and aperiodic regardless of graph connectivity, which guarantees convergence to a unique stationary distribution.*

*The damping factor d introduces a small uniform restart probability, ensuring ergodicity and preventing the random walk from being trapped in disconnected components.*

After the random walk process reaches a stable distribution, the stationary distribution $\pi^{*}$ is obtained, where each element $\pi_{i}^{*}$ represents the stationary probability of sample $u_{i}$. Since the transition matrix preserves strong similarities while suppressing weak connections, samples in densely connected regions receive higher stationary probabilities, while samples that deviate from the majority receive lower stationary probabilities, indicating a greater likelihood of being anomalous.

## 4. Robust Random Walk Based on Natural Neighbors for Outlier Detection Method

This section presents the proposed robust random walk based on natural neighbors for outlier detection (RWNOD) method. First, the overall RWNOD algorithm is introduced. Then, a detailed time complexity analysis is provided.

### 4.1. Outlier Detection Algorithm

The overall process of the proposed RWNOD method is summarized in Algorithm 2. The algorithm consists of two main stages: adaptive smoothing and robust random walk. First, natural neighbors are identified for each sample through an adaptive search process. Based on the natural neighbor relationships, the adaptive smoothing mechanism is applied to obtain smoothed positions, reducing local noise while preserving structural information. Next, a shadowed fuzzy similarity relation is constructed using the smoothed samples, where highly similar pairs are assigned full connection, highly dissimilar pairs are disconnected, and intermediate similarities are preserved. The transition probability matrix is then built by row normalization. Finally, a robust random walk with damping factor is performed to compute the stationary distribution, where the stationary probability of each sample directly serves as its anomaly score.
**Algorithm 2:** The RWNOD Algorithm
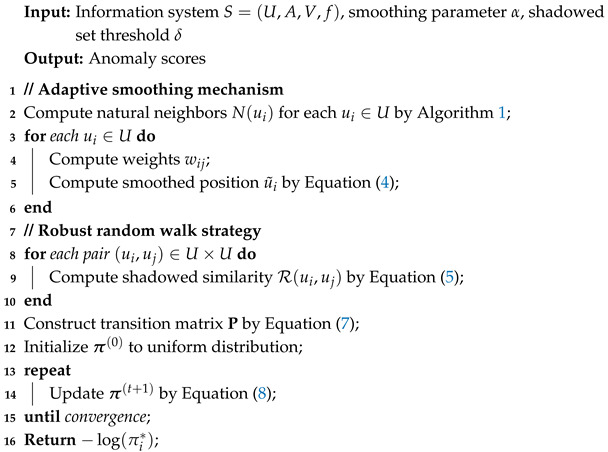


### 4.2. Time Complexity Analysis

The time complexity of the proposed RWNOD method is analyzed as follows. The natural neighbor search requires O(nlogn) time using a KD-tree structure, where *n* is the number of samples. The adaptive smoothing mechanism computes pairwise distances between each sample and its natural neighbors, which takes O(nk) time, where *k* is the average number of natural neighbors and typically k≪n. The construction of the shadowed similarity matrix requires computing pairwise distances among all *n* smoothed samples, which dominates the computational cost at $O(n^{2})$. The subsequent random walk iteration involves matrix-vector multiplications, each taking $O(n^{2})$ time, and the number of iterations is constant under a fixed convergence tolerance. Therefore, the overall time complexity of RWNOD is $O(n^{2})$.

## 5. Experiments

This section begins by describing the relevant settings for the experiments. Then, the performance test, statistical test, parameter sensitivity analysis, robustness test, ablation experiment, and interpretability analysis of the proposed outlier detection algorithm are carried out successively.

### 5.1. Experimental Settings

To evaluate the performance of the proposed method, experiments are carried out on twelve publicly accessible datasets. As summarized in Table 1, these datasets exhibit diverse characteristics: the number of attributes varies from 4 to 60, the sample size ranges from 107 to 6870, and the anomaly count spans from 6 to 176. All datasets are normalized using Min–Max scaling to map features into the [0,1] interval.

The proposed algorithm is compared with seven state-of-the-art outlier detection approaches. Table 2 lists the compared methods along with their hyperparameter tuning ranges, which are configured to achieve optimal performance. For each sample, an outlier score is produced to indicate its degree of deviation from normal patterns. Detection accuracy is assessed using the Area Under the ROC Curve (AUC), which summarizes the overall discriminative power, where values approaching 1 indicate superior detection capability.

### 5.2. Outlier Detection Performance Evaluations

**Figure 2:**
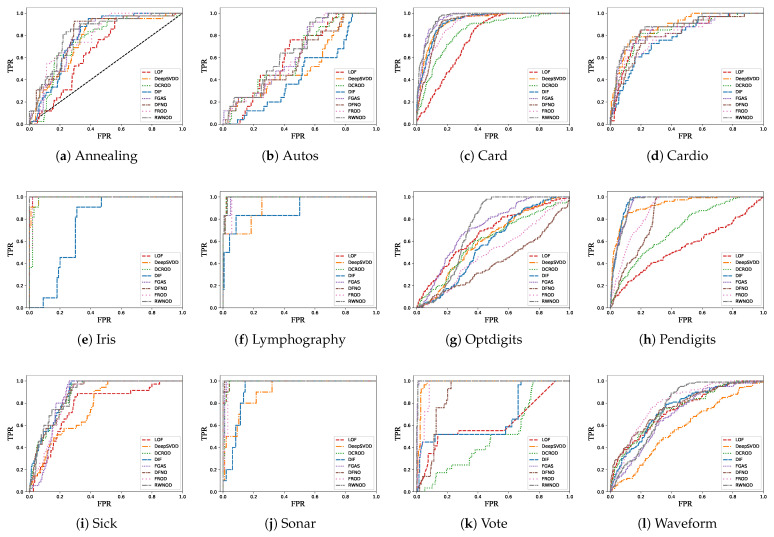
ROC curves for different datasets.

In this subsection, we employ ROC curves (Figure 2) to visually compare algorithm performance, followed by quantitative evaluations using the AUC metric. Table 3 reports the detailed AUC scores of all comparison methods on 12 datasets, where higher values indicate superior detection performance and the best results are highlighted in bold. RWNOD achieves the highest average AUC of 0.8841, outperforming all compared methods, and ranks first on 8 out of 12 datasets. These results indicate that the proposed adaptive smoothing mechanism and robust random walk strategy contribute positively to outlier detection, enabling the method to achieve competitive and robust performance across various datasets.

### 5.3. Statistical Analysis

In this subsection, we perform a statistical analysis of the experimental results. The Friedman test is first employed to examine whether there are significant performance differences among the compared algorithms. Subsequently, the Nemenyi test is conducted to further distinguish between them, and the resulting Nemenyi test figure visually illustrates the pairwise differences.

**Figure 3:**
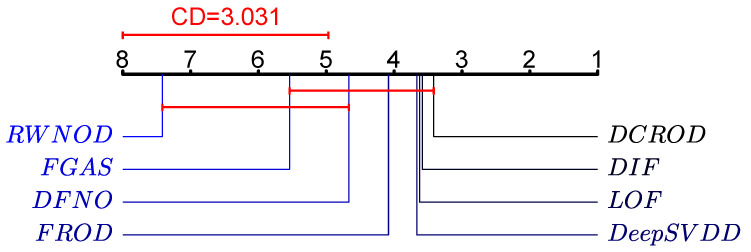
Nemenyi test figure on AUC.

As shown in Table 3, the Friedman test statistic is $\tau_{F}=5.08$, which exceeds the critical value of 2.13 at the α=0.05 significance level. This result rejects the hypothesis that all algorithms perform equally. The subsequent Nemenyi test with $CD_{0.05}=3.031$ (illustrated in Figure 3) indicates that RWNOD significantly outperforms DCROD, DIF, FROD, LOF, and DeepSVDD, while exhibiting no statistically significant difference from FGAS and DFNO.

### 5.4. Parameter Sensitivity Analysis

The proposed RWNOD method involves two key parameters: the smoothing parameter α and the shadowed set threshold δ. To investigate their impact on detection performance, we vary α from 0 to 1 with a step size of 0.05 (fixing δ=0.5) and vary δ from 0.5 to 1 with a step size of 0.05 (fixing α=0), respectively.

**Figure 4:**
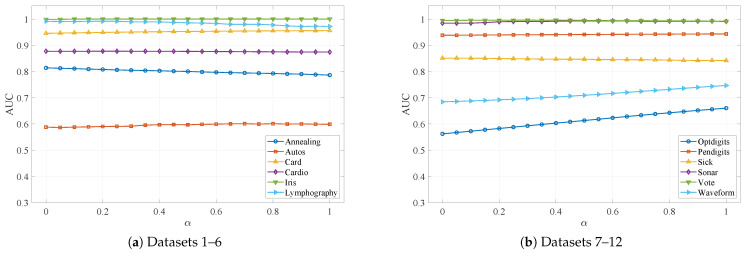
The variation of AUC with parameter α.

**Figure 5:**
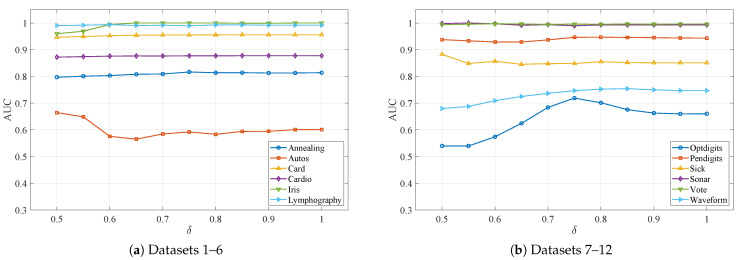
The variation of AUC with parameter δ.

As shown in Figure 4 and Figure 5, the AUC values exhibit discernible yet bounded variations across parameter configurations. For parameter α, the majority of datasets maintain relatively stable performance across different smoothing strengths, demonstrating that the adaptive smoothing mechanism consistently enhances local structure without introducing significant performance degradation. On a few datasets, performance gradually improves as α increases, indicating that stronger smoothing benefits certain data distributions by further suppressing local noise. For parameter δ, most datasets show stable AUC values across the threshold range, confirming that the shadowed set mapping reliably captures structural information. A small subset of datasets exhibits a unimodal pattern where performance peaks at intermediate δ values, suggesting that an appropriate threshold balances the trade-off between preserving strong connections and suppressing weak ones. Overall, while parameter selection can influence detection performance, RWNOD maintains competitive results across a wide range of configurations, demonstrating its robustness and practical applicability. Based on the above experimental results, we recommend α=0.8 and δ=0.75 as default values for practical applications, as they achieve stable and competitive performance across most datasets.

### 5.5. Attribute Noise Sensitivity Analysis

To assess the robustness of the proposed RWNOD algorithm under noisy conditions, we adopt the experimental strategy from references [35,38]. Specifically, we randomly select |U|×β samples, where β represents the noise level ranging from 0.1 to 0.5 with a step size of 0.1. For each selected sample, one attribute is randomly chosen, and its original value is replaced by a uniformly distributed random number within the attribute’s value range.

**Figure 6:**
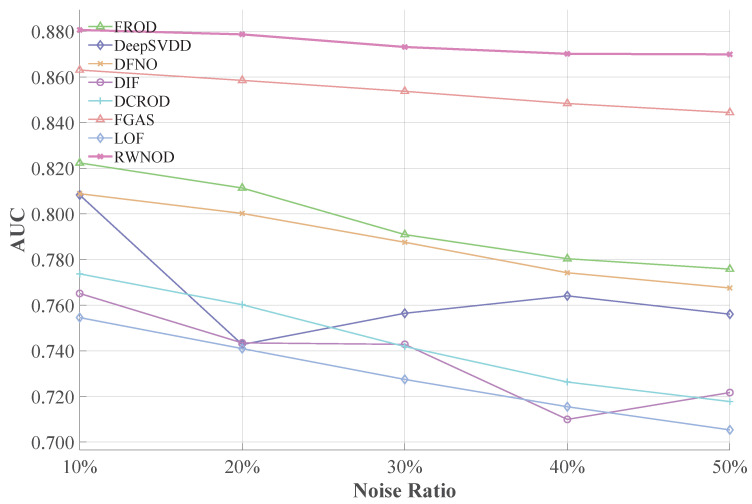
The variation of AUC on different noise levels.

Figure 6 presents the average AUC across all datasets as the noise intensity increases. The results demonstrate that RWNOD exhibits the most stable performance among all compared methods, with the smallest performance degradation under increasing noise levels. Meanwhile, RWNOD consistently maintains a clear performance advantage over other competing methods across varying noise intensities, confirming its superior robustness and reliability in noisy environments.

### 5.6. Ablation Experiment

To systematically evaluate the contributions of key components in the proposed RWNOD algorithm, we design a targeted ablation study with the following module replacement strategies:

- **Adaptive smoothing mechanism** replaced with no smoothing (i.e., raw data directly used for random walk).
- **Robust random walk strategy** replaced with standard random walk using Gaussian similarity without shadowed set mapping.

As shown in Table 4, the complete model (with both adaptive smoothing mechanism and robust random walk strategy) achieves the best AUC values on 11 out of 12 datasets, with an average AUC of 0.8841, significantly outperforming the other three variants. Removing the adaptive smoothing mechanism leads to a drop in average AUC to 0.8679, a decrease of approximately 1.62%. Removing the robust random walk strategy results in an average AUC of 0.8692, a decrease of about 1.49%. When both modules are removed, the average AUC further declines to 0.8526, representing a reduction of 3.15%. These results demonstrate that both components contribute positively to the overall performance and exhibit a synergistic effect. Meanwhile, the effectiveness and necessity of the proposed adaptive smoothing mechanism and robust random walk strategy are validated.

### 5.7. Interpretability Analysis

To demonstrate the interpretability of the proposed RWNOD method, we conduct a case study on the Sonar dataset. Table 5 presents the top 20 samples ranked by anomaly score, along with their stationary probabilities and the proportions of similarities falling into the three regions of the shadowed set mapping.

We take sample #106, which receives the highest anomaly score (5.992) and is correctly identified as an outlier (true label = 1), as a representative example for interpretability analysis. The results show that this sample has 96.2% of its similarities falling into the high-similarity region (t≥δ), and only 1.9% in the low-similarity region (t≤1−δ). This indicates that most of its connections to other samples are preserved as reliable edges, giving it a moderate stationary probability that still yields a high anomaly score.

Interestingly, we observe that sample #22 (true label = 0), ranked 11th, exhibits 73.6% in the high-similarity region and only 11.3% in the low-similarity region—values that might appear “normal” at first glance. However, the stationary probability of sample #22 is $4.796\times 10^{-3}$, slightly lower than that of sample #97 ($4.748\times 10^{-3}$), which is ranked 10th and has 60.4% in the high-similarity region. This subtle difference in stationary probability, influenced by the global structure of the graph, places sample #22 just behind the anomalous samples.

These observations confirm that the shadowed set mapping effectively distinguishes outliers from normal samples by adjusting the connectivity structure. Users can examine the three-region distribution of any given sample to understand why it receives a particular stationary probability, and consequently, why it is flagged as an outlier or not.

## 6. Conclusions

This paper develops a novel unsupervised outlier detection method by integrating adaptive smoothing mechanism and robust random walk strategy. First, an adaptive smoothing mechanism is proposed to leverage natural neighbors to actively adjust sample positions, reducing local noise while preserving structural information. Then, a robust random walk strategy is introduced to incorporate shadowed sets into the transition matrix, preserving reliable connections while suppressing unreliable ones for anomaly assessment. Finally, a corresponding outlier detection algorithm is developed, i.e., the RWNOD algorithm. Extensive experimental results demonstrate that our RWNOD algorithm not only achieves superior detection performance but also maintains strong robustness. However, the method still has certain limitations. The computational cost of constructing the full similarity matrix is relatively high for large-scale datasets. In addition, the performance may be affected when dealing with extremely high-dimensional data. To address these limitations, our future work will focus on developing more efficient similarity approximation techniques, exploring granular-ball-based optimization to reduce computational complexity, and leveraging dimensionality reduction strategies to enhance scalability and applicability.

## Figures and Tables

**Table 1:** Experimental datasets.

No.	Dataset Name	Abbr.	Attributes	Objects	Outliers	Outlier Proportions
1	annealing_variant1	Annealing	38	798	42	5.26%
2	autos_variant1	Autos	25	205	25	12.20%
3	cardio	Card	21	1831	176	9.61%
4	cardiotocography_2and3_33_variant1	Cardio	21	1688	33	1.95%
5	iris_Irisvirginica_11_variant1	Iris	4	111	11	9.91%
6	lymphography	Lymphography	18	148	6	4.05%
7	optdigits	Optdigits	64	5216	150	2.88%
8	pendigits	Pendigits	16	6870	156	2.27%
9	sick_sick_35_variant1	Sick	29	3576	35	0.98%
10	sonar_M_10_variant1	Sonar	60	107	10	9.35%
11	vote_republican_29_variant1	Vote	16	296	29	9.80%
12	waveform_0_100_variant1	Waveform	21	3443	100	2.90%

**Table 2:** Comparison algorithm.

No.	Algorithms (Year)	Descriptions	Time Complexities	Hyperparameter	Step
1	LOF (2000) [32]	Local Outlier Factor	$O(|C||U{|}^{2})$	k∈[2,60]	1
2	DeepSVDD (2018) [33]	Deep Support Vector Data Description	✘	✘	✘
3	DCROD (2022) [34]	Directed density ratio Changing Rate-based Outlier Detection	O(|C||U|log|U|)	k∈[2,60]	1
4	DIF (2023) [35]	Deep Isolation Forest for anomaly detection	O(|C||U||t·r|)	✘	✘
5	FGAS (2023) [24]	Fuzzy Granular Anomaly Score	$O(|C||U{|}^{2})$	σ∈[0.1,1]	0.1
6	DFNO (2025) [36]	Detecting Fuzzy Neighborhood Outliers	$O(|U{|}^{2})$	k∈[2,60]	1
7	FROD (2026) [37]	Fast and Robust Outlier Detection	$O\left(|C|\cdot (|U|+|GB{|}^{2}+|GB|\log|U|+k|U|)\right)$	k∈[2,60]	1
8	RWNOD (Ours)	Robust random walk based on natural neighbors for outlier detection	$O(n^{2})$	α∈[0,1], δ∈[0.5,1]	0.05, 0.05

**Table 3:** Experimental results on AUC.

Data	FROD	DeepSVDD	DFNO	DIF	DCROD	FGAS	LOF	RWNOD
Annealing	0.7925	0.7662	0.7997	0.7873	0.7552	0.7875	0.6671	**0.8168**
Autos	0.6278	0.5438	0.5927	0.4540	0.6189	0.6396	0.6282	**0.6646**
Card	0.9044	0.9238	0.9142	0.9269	0.8336	0.9527	0.7728	**0.9560**
Cardio	0.8113	**0.8780**	0.8095	0.7825	0.8344	0.8505	0.8400	0.8776
Iris	0.9909	0.9927	**1.0000**	0.7436	0.9827	**1.0000**	0.9982	**1.0000**
Lymphography	0.9812	0.9272	0.9930	0.8932	**0.9941**	0.9894	**0.9941**	**0.9941**
Optdigits	0.5002	0.6104	0.4065	0.5754	0.6019	0.7183	0.6668	**0.7190**
Pendigits	0.8798	0.9277	0.8338	**0.9526**	0.7118	0.9458	0.5506	0.9470
Sick	0.8573	0.7678	0.8768	**0.8848**	0.8750	0.8550	0.7669	0.8829
Sonar	0.9814	0.9103	0.9990	0.9186	0.9887	0.9938	0.9938	**1.0000**
Vote	0.9571	0.9804	0.8764	0.6707	0.5072	0.9952	0.6200	**0.9972**
Waveform	**0.7955**	0.5751	0.7661	0.7488	0.7433	0.6962	0.7412	0.7542
Average	0.8399	0.8170	0.8223	0.7782	0.7872	0.8687	0.7700	**0.8841**

**Table 4:** Ablation experiment results comparison table.

Adaptive Smoothing Mechanism	✓	✗	✓	✓
Robust Random Walk Strategy	✓	✓	✗	✓
Annealing	**0.8168**	**0.8168**	0.8139	0.8139
Autos	**0.6646**	0.6464	0.6009	0.5878
Card	**0.9560**	0.9464	**0.9560**	0.9458
Cardio	**0.8776**	**0.8776**	0.8775	0.8774
Iris	**1.0000**	**1.0000**	**1.0000**	0.9991
Lymphography	**0.9941**	**0.9941**	0.9918	0.9918
Optdigits	**0.7190**	0.6186	0.6603	0.5622
Pendigits	**0.9470**	0.9418	0.9435	0.9383
Sick	**0.8829**	**0.8829**	0.8510	0.8510
Sonar	**1.0000**	0.9887	0.9928	0.9845
Vote	0.9972	**0.9977**	0.9960	0.9947
Waveform	**0.7542**	0.7039	0.7468	0.6844
Average	**0.8841**	0.8679	0.8692	0.8526

**Table 5:** Top 20 ranked anomaly samples with shadowed set region distribution on Sonar.

Rank	ID	Score	Stationary Pro.	t≥δ (%)	1−δ<t<δ (%)	t≤1−δ (%)	Label
1	106	5.992	2.499$\;\times \;10^{-3}$	96.2	2.8	1.9	1
2	105	5.800	3.028$\;\times \;10^{-3}$	93.4	3.8	3.8	1
3	104	5.798	3.035$\;\times \;10^{-3}$	93.4	3.8	3.8	1
4	103	5.707	3.323$\;\times \;10^{-3}$	80.2	15.1	5.7	1
5	102	5.610	3.660$\;\times \;10^{-3}$	73.6	22.6	4.7	1
6	101	5.572	3.801$\;\times \;10^{-3}$	72.6	21.7	6.6	1
7	100	5.565	3.831$\;\times \;10^{-3}$	72.6	21.7	6.6	1
8	98	5.491	4.123$\;\times \;10^{-3}$	69.8	22.6	8.5	1
9	99	5.439	4.346$\;\times \;10^{-3}$	65.1	28.3	7.5	1
10	97	5.350	4.748$\;\times \;10^{-3}$	60.4	21.7	18.9	1
11	22	5.340	4.796$\;\times \;10^{-3}$	73.6	16	11.3	0
12	19	5.266	5.166$\;\times \;10^{-3}$	59.4	17	24.5	0
13	1	5.187	5.589$\;\times \;10^{-3}$	54.7	18.9	27.4	0
14	2	5.170	5.685$\;\times \;10^{-3}$	50.9	27.4	22.6	0
15	44	5.084	6.197$\;\times \;10^{-3}$	54.7	30.2	16	0
16	91	4.983	6.854$\;\times \;10^{-3}$	23.6	57.5	19.8	0
17	65	4.896	7.480$\;\times \;10^{-3}$	34	31.1	35.8	0
18	64	4.866	7.703$\;\times \;10^{-3}$	33	29.2	38.7	0
19	66	4.827	8.014$\;\times \;10^{-3}$	30.2	30.2	40.6	0
20	67	4.820	8.067$\;\times \;10^{-3}$	29.2	31.1	40.6	0

## Data Availability

The original contributions presented in this study are included in the article. Further inquiries can be directed to the corresponding author.
